# Supplementary material for: Reference values of urinary metabolites of organophosphate in healthy Iranian adults
Source: PLoS One. 2025 Jun 11;20(6):e0325037. doi: 10.1371/journal.pone.0325037 (PMC12157253; doi:10.1371/journal.pone.0325037)
Supplement: S1 Table — (DOCX) [file pone.0325037.s001.Docx]

| SC number | Provinces | Selected Province | Provincial capital | weight | | Total clusters | | | Total samples |
| --- | --- | --- | --- | --- | --- | --- | --- | --- | --- |
| 1 | Fars, Hamadan, Kurdistan, Lorestan, Markazi, Semnan, and Yazd | Yazd | Yazd | 0.17 | | 22 | 110 | | |
| 2 | East Azerbaijan, Gilan, Kerman, Razavi Khorasan, and Khuzestan | Khorasan Razavi | Mashhad | 0.26 | 34 | | | 170 | |
| 3 | Isfahan, Mazandaran, and Tehran | Tehran | Tehran | 0.32 | 42 | | | 210 | |
| 4 | Chahar Mahaal and Bakhtiari, Ilam, Kermanshah, and Kohgiluyeh and Boyer-Ahmad | Kermanshah | Kermanshah | 0.06 | 8 | | | 40 | |
| 5 | Bushehr, North Khorasan, South Khorasan, Qom, and Sistan and Baluchistan | Sistan and Baluchistan | Zahedan | 0.07 | 10 | | | 50 | |
| 6 | Ardabil, West Azerbaijan, Golestan, Hormozgan, Qazvin, and Zanjan | Ardabil | Ardabil | 0.12 | 16 | | | 80 | |
|  | **Total** | | | 1 | 132 | | | 660 | |

Table S1: Descriptive statistics on super-clusters (SCs), clusters and the assigned number of samples
