# Supplementary material for: Reference values of urinary metabolites of organophosphate in healthy Iranian adults
Source: PLoS One. 2025 Jun 11;20(6):e0325037. doi: 10.1371/journal.pone.0325037 (PMC12157253; doi:10.1371/journal.pone.0325037)
Supplement: Attachment — Submitted filename: PONE-D-24-11112.docx [file pone.0325037.s003.docx]

**The article reported metabolites of specific pesticides among 490 healthy Iranian adults to established reference values. Please, see my comments below.**

1. **The pesticides measured in this study include chlorpyrofos TCP, Diazinon, and Malathion. Are these four organophosphorus pesticides the most commonly used in Iran? In addition are there other pesticides and why are they not reported here. Furthermore, metabolites identified the only ones observed?**
2. **In the abstract the authors clearly state what the objective of the study is**
3. **#94: what is large quantities? Are you inferring that exposure to low levels do not safe?**
4. **There is the need to improve the language at several places in the text (e.g., #98-113)**
5. **#117: which definition?**
6. **#118-119 not clear**

**Methods**

1. **how can eligibility criteria help achieve representativeness. It's your sample strategy. Please, provide a brief description of your sample, sample size calculation, and data collection. I am concern about the eligibility criteria. I, For comparison among/between studies can you explain why adult was defined here as 25+ , (ii) the inclusion criteria not exposed to second hand smoke is a bit strange. How will you ensure this. Just inquiring about this with a questionnaire is not enough. Besides, it’s now established that combustion of biomass for cooking and heating produces similar chemicals as found in cigarette smoke. What about those exposed to household air pollution, (ii) what is your definition os healthy and how will you ensure this, (. iv) I find your eligibility criteria as too arbitrary. And also the one on occupational exposire**
2. **Provide detailed description on how the data was collected, by who and when?**
3. **The information provided on urine sampling is woefully inadequate**
4. **A bit of clarification is needed here about the exact instructions that were given to participants concerning the urine collection. The explanation should clarify for males and females.**
5. **Provide soe detail information about the questionniare and its administration in the text**
6. **provide a detail description of the analysis and the models that were used**

**Results**

1. **were females over-sampled? since the purpsoe of this study was to generate a reference for the iranian population, is the current population representative enough?**
2. **one of the serious flaws of this study was selection bias and not too sure if this study is suitable for the development of a reference**

**Discussions**

1. **discuss the reasons for the differences between communities. Else, it will appear as if the results are repeated**
2. **#291: what type of milk?**
3. **The authors should carefully discuss their results**
4. **The strength and limitations of thr study should be thoroughly discussed in the light of selection bias, information bias and confounding where possible**
