# Supplementary material for: Reference values of urinary metabolites of organophosphate in healthy Iranian adults
Source: PLoS One. 2025 Jun 11;20(6):e0325037. doi: 10.1371/journal.pone.0325037 (PMC12157253; doi:10.1371/journal.pone.0325037)
Supplement: Attachment — Submitted filename: response to reviwers 5Nov.docx [file pone.0325037.s005.docx]

Please use the space provided to explain your answers to the questions above. You may also include additional comments for the author, including concerns about dual publication, research ethics, or publication ethics. (Please upload your review as an attachment if it exceeds 20,000 characters)

Reviewer #1

The author may revise the manuscript with proper scientific evidence and supporting data to arrive at definite conclusions. The reasons are to be clearly defined and stated in the conclusions part.

**Response**: Thanks for this comment. As the reviewer asked correctly, we organized some sections of discussion and added subheadings for better understandability of the text in the discussion section.

Reviewer #2

Dear authors, I read your manuscript"Reference Values of Urinary Metabolites of Organophosphate in Healthy Iranian Adults"
To improve the article's quality, I recommended the following comments:

1. In the materials and method section, the type of metabolites and their properties such as purity have not been written.

**Response**: Thanks for this constructive comment by the reviewer. Corrected

2. The name of the internal standard has not been mentioned. Please add the name of the internal standard along with a reference.

**Response**: Thanks for your recommendation. We added it to the QA/QC section of the paper . Standard solutions were prepared using Pesticide Mix 1510 (Sigma Aldrich Company) at a concentration of 50 ppm, which included the pesticides Diazinon, Malathion, and Chlorpyrifos. From this mix, standards at concentrations of 20, 50, 100, and 200 ppb were prepared and analyzed in the LC-MS device in the same volume as the samples. Isotopically labeled Dialkyl Phosphates, including Diethyl Phosphate (DEP)-d10 and Dimethyl Phosphate (DMP)-d6, were used as internal standards.

3. What do you mean by reference value? About reference value, I think that you should cite to new references. References of 17, 18, and 19 for this keyword are very old. What is the difference between reference dose and reference value?

**Response**: Thank you for this comment. We have updated the references .The definition of the reference value of the German [HBM](https://www.sciencedirect.com/topics/earth-and-planetary-sciences/biomonitoring) Commission – RV95 – is the 95th percentile of the substance of interest at a specific time point rounded off within its 95% CI and agrees with the International Union of Pure and Applied Chemistry (IUPAC) guideline. The choice of the 95th percentile and 95% CI can be motivated by the convention in hypothesis testing where the 5% most extreme sample values indicate unusual values. But the A reference dose is “an estimate (with an uncertainty spanning perhaps an order of magnitude or greater) of a daily exposure level for the human population, including sensitive subpopulations, that is likely to be without an appreciable risk of deleterious effects” for chronic exposures.

4. Please cite new references for all materials and methods subtitles. Almost, more of them are without reference.

**Response**: Thanks for this constructive comment by the reviewer, we corrected this section according to your comments.

5. In the results section, numerical data lack unit. please check all of them.

**Response**: Thank you for this comment, Yes, we corrected that

6. All references are very old. I think it is better to discuss new references. The discussion section is written poorly.

**Response**: Thanks for this comment. As the reviewer asked correctly, we organized some sections of discussion and added subheadings for better understandability of the text in the discussion section.

7. Results are very poor. Except for Table 7( Visceral fat ), other results are without any significant relationship.

**Response**: Thank you for this comment. As this is a nationwide study, non-significant results are also important for policymakers and other researchers. Moreover, this study can pave the way for other researchers to know the effect of each variable on results. The main goal of this study was to evaluate reference values of urinary metabolites of organophosphate in healthy Iranian adults.

8. I think that the conclusion could be written better.

**Response**: We want to deeply thank the reviewer for this comment. We added better healthcare and policy implications of our findings in order to better elucidate the needs and future research.

Reviewer #3

This manuscript presents a study on the urinary levels of organophosphate pesticide metabolites among a healthy Iranian adult population. The study provides valuable data on exposure levels across different regions and demographic groups within Iran. However, the manuscript requires significant revisions to meet the publication standards.

Title
Make the title more descriptive and specific about the study's focus, such as "Assessment of Organophosphorus Pesticide Exposure Among Iranian Adults Using Urinary Biomarkers."

Abstract
Abstract does not cover the main aim of the study which is derivation of reference values (RV95s). why this is not reported? Ensure the abstract is concise and free from redundancy. Simplify complex sentences and use straightforward language to enhance readability.
Define any abbreviations or acronyms.
Clarify the results. For instance: Metabolite levels ranged from 0-24.9 µg/l for TCP (median: 2.8 µg/l, 2.9 µg/gcrt….
What 0 means in the range? Below detection limit? Please clarify.

**Response**: Thanks for this constructive comment by the reviewer. We corrected all points according to your comments.

Introduction
- The text adequately highlights the importance of the issue but could be more specific about the context of Iran (a more detailed explanation of the current state of pesticide use and exposure in Iran), and the gap in biomonitoring data specific to this region.

**Response**: Thanks for this constructive comment by the reviewer. We addressed this in the introduction.

- Expand the literature review to include more recent studies and data on pesticide exposure and its health effects, particularly those relevant to the Iranian context.

**Response**: Thanks for this constructive comment by the reviewer. We addressed this in the introduction.

The introduction lacks citations in some areas, and some references are not formatted correctly (e.g., "(10())").

**Response**: Thanks for this constructive comment by the reviewer. Yes, we corrected that.

- Clearly state the study's objectives at the end of the introduction. This helps to provide a clear direction for the paper.

**Response**:We want to deeply thank the reviewer for this comment. We added it to the last paragraph of the introduction

Please revise the line 115-119 as following:

“ In Iran, the use of pesticides has increased significantly over the last decade, raising serious concerns about their toxicological and detrimental effects. Despite this, there has been no national biomonitoring study or reported RV95 for pesticides in Iran. This study aims to fill this gap by calculating RV95s using data from an HBM study that measured pesticide metabolites in urine and examined effective demographic factors in Iran.”

**Response**: Thanks for this constructive comment by the reviewer .we changed the last paragraph to:

However, in Iran, despite a sharp increase in pesticide use over the last decade, there is a notable absence of national biomonitoring data and no reported RV95 values specific to the Iranian population. This gap in data raises significant concerns about the toxicological and health impacts of pesticide exposure in Iran. This study aims to address this critical gap by calculating RV95s for pesticide metabolites using data from an HBM study that analyzed urine samples and examined key demographic factors in Iran.

-The introduction lacks clear structure and flow, with some sentences being long and complex, making it difficult to follow. The transition between general information and the specific focus of the study is not smooth. Some examples:
Please revise the sentence line as: “The annual use of pesticides in agriculture continues to rise due to the expansion of agricultural activities”
And the following sentence as “ While pesticides are essential tools in modern agriculture for improving food supply, protecting crops, and controlling pests and disease vectors, their widespread use poses significant risks to human health and the environment”.

**Response**: Thanks for this constructive comment by the reviewer We revised the introduction section thoroughly and tried to address all the comments.

Material and method

Age range of the participants should be mentioned.

**Response**: Thank you for your comment. The age range of the participants in the study described in the article is **25 years or older.** The age range is added to the Material and Method section.

The methods section briefly mentions that "after data cleaning, 490 samples were used for statistical analysis." There are no details on the specific data cleaning procedures (checking for outliers, inconsistencies, duplicates, and invalid data entries) or criteria used to determine which samples were included or excluded (Samples with incomplete demographic data or urine samples with insufficient volume). How is the data cleaning process done? How did you handle the missing information? Describe the methods used for imputing missing data and justify the choice of imputation method.

**Response**: The sampling design was developed based on a calculated sample size of 383, assuming a 50% baseline prevalence for urinary toxic metal(loid) levels, as no prior national surveys had assessed these parameters. Using Cochran's formula for large populations (Cochran 2007) and accounting for a 1.5 design effect (Salganik 2006) and an 85% response rate (Dey 1997), the sample size was adjusted to 660 across 132 clusters. Clusters were chosen via model-based clustering (Banfield and Raftery 1993; Scrucca et al., 2016), allowing us to organize Iranian provinces into six super-clusters (SCs) based on covariates such as particulate matter (PM10), pesticide use, and rates of cancer and chronic respiratory diseases. The capitals of these provinces—Ardabil, Kermanshah, Mashhad, Zahedan, Tehran, and Yazd—served as essential sampling units, with cluster coordinates within urban zones generated for systematic sampling (Bivand et al., 2019). Supplementary Table 1 provides details on each SC, cluster, and assigned sample size and inclusion and exclusion criteria.

Using the DIGIT data collection platform, ensured enough meta data to avoid duplication and data loss. This platform uses electronic questionnaires and multiple validation measures such as mandatory questions and limiting the answers to multiple choice to enhance data quality. An invalid data entry was impossible as the validations for each question and the answers were in place at the time of interviewing. However from 660 samples the ones without sufficient urine samples where excluded from data analysis (how many?).

The study incorporates **non-response weights** to adjust for participants who may not complete the full procedure (including questionnaires, anthropometry). There are **nine adjustment weights** to account for various types of non-responses, such as questionnaire-based non-response and laboratory-specific non-response for different sources (air, food, water, and urine). Any missing data in this was handled by using imputing average values for demographic data.

Line 135-137: delete this sentence. It does not belong here.
Provide the durations at various temperatures to ensure sample integrity.
Information on validation procedures for analytical methods, including recovery rates, precision, accuracy, are missing.
I could not find use of quality control samples to monitor method performance. Please elaborate on descriptions of how calibration curves are validated and the specific acceptance criteria for QC sample
Line 165 remove “ 200”: The limit of detection 165 (LOD) and limit 200 of quantification (LOQ) for these compounds are indicated in table 2.

**Response**: Thank you for your insightful question regarding the use of quality control (QC) samples in monitoring method performance. Calibration curves and quality control (QC) samples are essential components in validating analytical methods, particularly in LC-MS analysis of pesticides. A calibration curve establishes the relationship between known concentrations of an analyte and the instrument's response, allowing for the estimation of unknown concentrations in test samples. Calibration standards are prepared from a reliable stock solution, and the relationship is evaluated using linear regression analysis, with the correlation coefficient (r) ideally >0.995. According to regulatory guidelines, the accuracy of back-calculated concentrations from the calibration curve should be within ±15% of the nominal concentration for all levels except for the lower limit of quantification (LLOQ), which should be within ±20%, with at least 75% of the calibration standards meeting these criteria. QC samples are critical for monitoring the performance of the analytical method, with accuracy within ±15% of the nominal concentration for all levels except LLOQ, which should be within ±20%, and the coefficient of variation (CV) for QC samples at each concentration level ideally ≤15%, ensuring that an analytical run is only accepted if both calibrators and QCs meet their respective acceptance criteria.

Line 182: what do you mean with “physical measurements”? Anthropometry?
was any sensitivity analysis conducted to assess the impact of for example different cofounders or missing data on the study results?
Lots of grammatical error and long and complex sentences. Please revise the text.

**Response:** Thank you for your valuable comment. Physical measurements are height and weight plus assessing the visceral fat, muscle percentage, body fat. The physical measurements were assessed for having an impact on the results of the study. However, the findings were insignificant therefore not reported.

Method for calculating of RV is missing.

**Response**: Thank you for your comment. We explained about RV95 in the introduction and Material and Method section.

The entire manuscript was revised for grammatical errors and English structure.

**Response**: Thanks for this good comment by the reviewer. We revised the entire article in terms of writing

While the importance of lifestyle factors, or food patterns or other factors in interpretation of the results is mentioned by the author in the introduction section but it seems no data on these aspects is collected in this study. This is one of the limitations of the study that should be mentioned in the discussion.

**Response**: Thanks for this constructive comment by the reviewer. We added that to the limitation section, as the reviewer asked (highlighted): “Then, the effect of lifestyle factors, food patterns, and other influential factors were not assessed in the present investigation which highlights the need for further exploration of these associations.”

Results
The results are comprehensive but somewhat difficult to follow due to the dense text and lack of clear subsections. Clear headings and subheadings would improve readability.

**Response**: Thank you for this valuable comment which increased the readability of the results section. We added appropriate subheadings to the results section for better readability. The added subheadings are “Baseline characteristics,” “Anthropometric indices of men and women,” “Urine pesticide levels in the Iranian population,” and “The relationship between pesticide level and demographic features.”

The descriptive statistics for anthropometric measurements and body composition are well-presented. However, including more comparative statistics (e.g., t-tests or ANOVAs) could provide insight into whether these differences are statistically significant.
**Response**: I want to thank the respected reviewer for this great comment. The main goal of this study was to provide reference values for pesticide levels in the Iranian population. Thus, we focused our study on the association between anthropometric measurements and pesticide levels. To improve the readability and make the study more useful and concise, no statistical tests were conducted between different groups in anthropometric measurements.

Line 200- revise as following:

“ The study population was predominantly female (58.09%). However, in the age group 55-65, the gender distribution was equal”

**Response:** Thank you for this awesome comment. We changed the mentioned sentence as the respected reviewer mentioned.

Revise line 204 “ Men were taller and weighed more than women, whereas women had higher BMI and body fat percentages. Detailed statistics are provided in Table 4.”
It is mentioned that the population in the study broken down into five quintiles, from the income/wealth ladder perspective. But the method used, and rationale is not mentioned in the method section. Question is why this approach is taken while relationship between pesticide concentrations in urine and this factor is not assesses.

**Response**: Thank you for this comment. We changed the mentioned sentence as the respected reviewer mentioned.

Discussion:
The discussion disproportionately focuses on chlorpyrifos, with less detailed analysis of diazinon and malathion. A more balanced discussion would provide a comprehensive understanding of all three pesticides.
**Response**: Thanks. In fact, the other two organophosphate agents were less investigated in the literature, compared to chlorpyrifos. However, we added a discussion on them to better elucidate the current situation (highlighted): “The other two reported pesticides in our study were diazinon and Malathion which were less investigated in the literature, compared with chlorpyrifos. Diazinon is another organophosphorus insecticide that is classified as a moderately hazardous compound (class II) based on the World Health Organization (WHO) classification system (35). There are a limited number of studies that have assessed diazinon levels in human subjects. In a small study on five subjects, Garfitt et al. showed that urinary dialkyl phosphate levels were highest between 2 and 12 hours after exposure to diazinon. Also, they showed that 60% of the oral dose and 1% of the dermal dose were excreted as urinary DAP metabolites (36). Malathion, also as another organophosphate insecticide, is used for mosquito-borne disease control such as West Nile disease, and in some cases as the treatment of head lice (37, 38). The difference between urine levels of these two compounds in six superclusters in Iran represents the need for regional decision-making regarding the use of these insecticides.”

Discussion on the potential source of exposure to these pesticides is missing. Did author collect any data on lifestyle and dietary habits?

**Response**: Thanks for this accurate comment by the reviewer. Lack of data on lifestyle factors and food patterns was a limitation of this study which was acknowledged in the limitation section (highlighted): “Then, the effect of lifestyle factors, food patterns, and other influential factors were not assessed in the present investigation which highlights the need for further exploration of these associations.”

Occupational exposure to pesticide is excluded however information on the job of Participants are missing.

**Response**: Thanks for this valuable comment, Following a previously published protocol (Soleimani et al., 2021), the 660 individuals selected for this study were (i) at least 25 years old, (ii) non-smoker and have no contact with smokers, (iii) apparently healthy and not pregnant, (iv) not take any medications within the last month, (v) not have a history of any chemotherapy or radiotherapy throughout their lifetime, (vi) **not exposed regularly to high-polluted environments at their work place. i.e., individuals’ occupation should not include casting, metallurgy, welding, and soldering, and they should not work at rubber, asphalt, soda, and pesticide manufacturing industries, or restaurant kitchens, or be a professional driver that might be exposed to road dusts**.

The discussion of non-significant findings, especially regarding demographic factors, is lacking. Providing potential explanations or considerations for these results is recommended.

**Response**: Thanks for this great comment. We agree with the reviewer on the need for discussion on non-significant findings. We added some data on them to better show and discuss the results: “The associations of demographic features and pesticide concentrations were also assessed, and the only significant finding was the correlation of body fat and BMI with chlorpyrifos levels. The lack of association between sex and age with these levels shows that a wide range of people is at risk of exposure to these agents and policies in Iran should cover all the population. Moreover, unlike many other exposures, there was no correlation between the wealth index and urine levels of Chlorpyrifos, diazinon, and malathion. Hence, interventions should be made irrespective of socioeconomic status, highlighting the need for general and inclusive policies.”

While comparisons with previous studies are made, more in-depth analysis and discussion of why differences might exist (e.g., differences in study populations, methodologies, and environmental factors) would be beneficial.

**Response**: Thanks for this comment by the reviewer which will certainly enhance the understandability of our discussion section. We provided hypotheses for these differences in the discussion section (highlighted): “Two studies performed investigation among termiticide applicators populations and as expected, they reported significantly higher urine levels, compared to ours, which were on the general population (27, 28).”, “The difference could be attributed to the differences in the location and occupation of the studied population.”, and “However, the overall numbers for the country and Mashhad supercluster in the current study both reported lower numbers in comparison with the mentioned study (1.13 and 0.41 μg/L compared to 1.3 μg/L, respectively). These minor differences could be mainly because of differences in measurement and populations.”

There is limited discussion on the potential biological mechanisms underlying the observed associations, particularly for the significant correlation between visceral fat and chlorpyrifos levels.

**Response:** Thanks. We added plausible mechanisms for this association to the discussion section, as the respected reviewer asked (highlighted): “The associations of demographic features and pesticide concentrations were also assessed, and the only significant finding was the correlation of body fat and BMI with chlorpyrifos levels. The underlying mechanism for this association might be the one observed previously in animals exposed to chlorpyrifos which later developed dyslipidemia and obesity (24, 25). The biological cause could be the promotion of 3T3-L1 adipocyte adipogenesis by chlorpyrifos and fat accumulation in addition to the inhibition of phosphorylation of adenosine 5′-monophosphate-activated protein kinase (AMPK) to promote the expression of these key adipogenic regulators (26).”

The discussion is somewhat disorganized, with some points repeated and others not clearly linked to the results. Clearer subheadings and a more structured approach would improve readability.

**Response**: Thanks for this comment. As the reviewer asked correctly, we organized some sections of discussion and added subheadings for better understandability of the text in the discussion section.

A more detailed evaluation of the study's limitations (including the cross-sectional design, potential biases in self-reported data, among others) and their potential impact on the results is required.

**Response**: Thanks for this comment. We added these limitations to the discussion (highlighted): “Finally, the cross-sectional design of the study and potential biases in self-reported data might contribute to the results observed and not only threaten the generalizability of the findings but also prevent us from deducing any causal association.”

Discussion on RV95 is limited. More detailed information and comparison with other available RVs is required. are there any HBMGVs for these pesticides? please add more information on interpretation of the results.

**Response**: Thanks for this comment regarding the discussion on RV95. We added a comparison with HBMGVs and the lack of data on that, hence, suggesting future studies on this. It is highlighted in the discussion section: “Third, this method (RV95) is merely a statistical model and cannot be applied to predict health effects. This makes RVs different from Human biomonitoring guidance values (HBM-GVs) which are important tools for the determination of population exposure to chemicals and as part of health-related risk assessments (46). Our study did not have HBM-GVs for the mentioned pesticides and since RVs are not suitable for assessment of a substance distribution, further studies aiming at finding HBM-GVs are warranted.”

Conclusion:
Expand the conclusion to discuss the broader public health implications of the findings and how they can inform future research and policy.
How this study filled the gap mentioned in the introduction?

**Response**: We want to deeply thank the reviewer for this comment. We added better healthcare and policy implications of our findings in order to better elucidate the needs and future research (highlighted): “Our findings provide a clear image of the current situation regarding pesticide exposure in Iran based on a clustering method representative of the whole Iranian population. Considering the high use of pesticides in Iran and the lack of enough literature on the reported levels of three main pesticides including Chlorpyrifos, diazinon, and Malathion, these studies could provide regional levels and cutoffs for policymakers in order to better design programs for the reduction of pesticide exposure in Iran. Future research should also focus on assessing other pesticides and provide a more comprehensive assessment of these organophosphates with a larger sample size, and calculation of HBM-GVs.”

Reviewer #4

The article reported metabolites of specific pesticides among 490 healthy Iranian adults to established reference values. Please, see my comments below.

1. The pesticides measured in this study include chlorpyrofos TCP, Diazinon, and Malathion. Are these four organophosphorus pesticides the most commonly used in Iran? In addition are there other pesticides and why are they not reported here. Furthermore, metabolites identified the only ones observed?

**Response:** We want to deeply thank the reviewer for this comment. Based on literature review, we selected the most used pesticides in Iran and measured their metabolites in urine.

2. In the abstract the authors clearly state what the objective of the study is
3. #94: what is large quantities? Are you inferring that exposure to low levels do not safe?
4. There is the need to improve the language at several places in the text (e.g., #98-113)
5. #117: which definition?
6. #118-119 not clear

**Response:** Thanks for reviewing the manuscript and providing valuable comments, we revised the introduction section thoroughly and tried to address all the comments.

Methods

1. how can eligibility criteria help achieve representativeness. It's your sample strategy. Please, provide a brief description of your sample, sample size calculation, and data collection. I am concern about the eligibility criteria. I, For comparison among/between studies can you explain why adult was defined here as 25+ , (ii) the inclusion criteria not exposed to second hand smoke is a bit strange. How will you ensure this. Just inquiring about this with a questionnaire is not enough. Besides, it’s now established that combustion of biomass for cooking and heating produces similar chemicals as found in cigarette smoke. What about those exposed to household air pollution, (ii) what is your definition os healthy and how will you ensure this, (. iv) I find your eligibility criteria as too arbitrary. And also the one on occupational exposure

**Response:** The eligibility criteria in the study play a key role in ensuring representativeness by focusing on creating a homogenous sample while avoiding bias from individuals with health conditions or high environmental exposures. It should be mentioned that the eligibility criteria were set to cover the whole project’s objectives which included the study of other pollutants as well as pesticides. There were two other main pollutants groups, metal(oids) and Poly Aromatic Hydrocarbons (PAHs).

(i) Adults were defined as those aged 25 years or older. This age criterion was likely set to capture a population with stable environmental and occupational exposures, which may be less variable than those under 25 who might have differing lifestyle factors or developmental considerations. Moreover, this aligns with other studies to maintain comparability.
(ii) The whole questionnaire was asked by an interviewer and no validation measure could be taken about ensuring no exposure to second hand smoke in the household. However, the study focuses on excluding participants exposed to secondhand smoke to minimize confounding from smoking-related pollutants like PAHs. At the end we wanted to avoid extra exposure to pollutant than everyday living method of normal Iranians.
(iii) No current chronic disease and use of medication. Going under chemotherapy in the past 5 years.
(iv) As mentioned on the point (i), many of the eligibility criteria were set to meet the project objectives on measuring exposure to Pesticides, PAHs, and Metal(oids).

2. Provide detailed description on how the data was collected, by who and when?

**Response:**  **Data Collection Overview**:

- The data was collected as part of a cross-sectional study aimed at measuring exposure to environmental pollutants, including heavy metals, polycyclic aromatic hydrocarbons (PAHs), and pesticides. The pollutants were measured in various matrices such as air, food, water, and urine.

 **Sample Size and Sampling Strategy**:

- The study involved 660 households from 132 clusters across six super-clusters (SCs) representing urban areas in Iran. From which 490 subjects entered the final analysis. The sample size was calculated using Cochran’s formula, adjusted for response rates and design effects to ensure the sample's representativeness.

 **Data Collection Methods**:

- **Questionnaires**: Data collection began with administering several validated questionnaires, including demographic, air quality, anthropometry, and food frequency questionnaires (FFQ).
- **Duplicate Portion Sampling (DPS)**: Participants were required to prepare duplicate portions of their daily food intake over three consecutive days. These samples were collected and analyzed to determine dietary intake of heavy metals, pesticides, and PAHs
- **Air, Water, and Urine Samples**: Samples were collected from participants' households over a four-day period. Morning urine samples were collected, and air quality samples were taken from each participant's home to measure indoor pollutants

 **Who Collected the Data**:

- Data collection was conducted by trained field staff supervised by local university teams. The Tehran University of Medical Sciences and the Non-Communicable Diseases Research Center (NCDRC) coordinated and monitored the study. Field supervision included both online and in-person oversight to ensure accuracy in sample collection

 **When Data Was Collected**:

- The study was carried out as part of a national population health survey, Sampling was done from 1/5/2019 to 15/3/2021 on Iranian adults aged 25 and above.

3. The information provided on urine sampling is woefully inadequate

**Response**: Thank you. More explanation is added to the manuscript.

4. A bit of clarification is needed here about the exact instructions that were given to participants concerning the urine collection. The explanation should clarify for males and females.

**Response**: Participants were asked to collect morning urine samples on the fourth sampling day. The collected samples needed to be stored at 4°C in cooling boxes during transport to ensure preservation until laboratory processing. The samples were separated into aliquots and stored at -20°C for a few days, before transferring to the laboratory or -80°C storage units for long-term storage. Explicit instructions in the form of a leaflet were given to all participants about how to take the urine sample per the study Standard Operating Procedure. Female participants were asked for certain way of wash before sampling in the time of menstruation.

5. Provide soe detail information about the questionniare and its administration in the text

**Response**: Thank you, the information is added to the text.
6. provide a detail description of the analysis and the models that were used

**Response**: Thank you for your comment. The data analysis in this study was done using a combination of descriptive statistics, sampling weights, and modeling techniques tailored to different pollutants.

1. **Descriptive Analysis**:
   - Descriptive statistics (mean, standard deviation) were calculated for demographic, anthropometric, and laboratory variables. These measures were used to present baseline data and overall pollutant concentrations across the study’s sample.
2. **Weighted Sampling Design**:
   - The analysis incorporated nine adjustment weights to account for non-response rates across different domains, such as demographic questionnaires, food frequency questionnaires, anthropometry, and lab-based variables (air, food, water, urine). This was crucial to making representative population estimates. The non-response weight was defined as the inverse of the non-response rate in each sampling cluster.
3. **Modeling Approaches**:
   - Gaussian Mixture Models were employed for clustering analysis to categorize provinces into six super-clusters based on particulate matter (PM10) levels, cancer incidence rates, and pesticide usage. This model-based clustering was used to determine sample allocation across regions.
   - The pollutant data from different sources (air, water, food, urine) were analyzed using generalized additive models and linear regression models to identify relationships between pollutant levels and variables like age, location, and wealth index.
4. **Statistical Significance**:
   - For all analyses, a p-value less than 0.05 was considered statistically significant.
5. **Quality Control**:
   - The study adhered to strict quality assurance (QA) and quality control (QC) protocols, including the use of international reference materials and analysis of blanks and spiked samples for accuracy. For instance, the limit of detection (LOD) was defined as three times the standard deviation of blank values.

This comprehensive approach ensured the robustness of the data analysis, from descriptive measures to advanced modeling techniques.

Results
1. were females over-sampled? since the purpsoe of this study was to generate a reference for the iranian population, is the current population representative enough?

Response: Thank you for this comment. The current study is a nationwide study involving all across Iran. In the sampling stage, we tried to make it nationwide representative. Although we tried our best, this study has some limitations that were discussed in the last paragraph of the discussion before the conclusions section.

2. one of the serious flaws of this study was selection bias and not too sure if this study is suitable for the development of a reference
Response: Thank you for this comment. In this study, we tried to reduce the selection bias by involving key cities in the study and making it a good representative of the current Iranian population. However, it still has some potential limitations that were discussed in the limitations paragraph.

Discussions
1. discuss the reasons for the differences between communities. Else, it will appear as if the results are repeated

**Response:** Thanks for reviewing the manuscript and providing valuable comments. We added possible reasons for differences among the similar studies to the discussion section. Moreover, we organized the discussion section: *“Two studies performed investigation among termiticide applicators populations and as expected, they reported significantly higher urine levels, compared to ours, which were on the general population (27, 28).”*, *“The difference could be attributed to the differences in the location and occupation of the studied population.”*, and *“However, the overall numbers for the country and Mashhad supercluster in the current study both reported lower numbers in comparison with the mentioned study (1.13 and 0.41 μg/L compared to 1.3 μg/L, respectively). These minor differences could be mainly because of differences in measurement and populations.”*

2. #291: what type of milk?

**Response:** Thanks. We wanted to mention “breast milk” which was corrected in the discussion: *“It was conducted on samples from mothers’ urine and breast milk.”*

3. The authors should carefully discuss their results

**Response:** Thanks for this constructive comment. We changed the discussion in terms of content and organization. The changes are highlighted throughout the discussion section.

4. The strength and limitations of thr study should be thoroughly discussed in the light of selection bias, information bias and confounding where possible

**Response:** Thanks for this comment. We added several limitations to our study including those mentioned by the reviewer. It is highlighted in the discussion section: *“Finally, the cross-sectional design of the study and potential biases such as selection bias, information bias, and confounding bias in addition to self-reported data might contribute to the results observed and not only threaten the generalizability of the findings but also prevent us from deducing any causal association.”*

Reviewer #5

The study focuses on the metabolites of Chlorpyrifos (TCP), Diazinon (IMPY), and Malathion (Malathion dicarboxylic acid). Key parameters recorded include age, gender, wealth index, and body composition metrics such as body fat, muscle, visceral fat, and BMI. Urine samples were collected and analyzed using LC-MS/MS, and the data were processed through descriptive statistics and multiple linear regression.
However there are some limitations:
a.Sample Size and Representativeness: This study included 490 people from six provinces in Iran. Iran has 31 provinces with different geographical environments and agricultural distributions. Is the selected population representative?please explain.

**Response:** Thanks for this comment and evaluation of our manuscript. The sampling method used in our study was modeled with clustering and each super-cluster was representative of several other clusters. The details of the methodology used in this study have been published in another article (doi: 10.1007/s11356-020-12004-3) which was cited for more information. We also added some explanations to the strengths and limitations section: *“Then, despite performing clustering and modeling, the sample might not be representative of all populations in 31 Iranian provinces.”* and *“This was the first nationally conducted study on the assessment of exposure to organophosphate pesticides in Iran (20). The model-based clustering designed by Banfield et al. and Scrucca et al. was used and implemented on representative sample size (46, 47). The methodology of clustering with the aim of being representative of the whole Iranian population is among the strengths.”*

b.Single-Time Urine Sample Collection: Urine samples were collected only once from each participant. Pesticide levels can fluctuate based on agricultural cycles, dietary changes, or seasonal variations.Urine samples should be collected three times on different days.

**Response:** Thanks for this great comment by the reviewer. The lack of temporal assessment in the analysis is one of the limitations that should be acknowledged. So, we added this as a limitation of our study (highlighted): *“Fifth, since the measurement of urine samples was performed once only, the fluctuations and variations in pesticide levels by agricultural cycles, dietary changes, or seasonal variations were not considered in the analyses, hence, affecting the results.”*

c.In disscusion,lack of recent references: please add the latest references and remodify discussion section.

**Response:** Thanks. We tried to update our references with the most recent published studies. However, since this has been an issue from many years ago, citing relevant original studies published before 2010 was inevitable.

d.The English in this article is not academic or standard enough. the language should be polished.

**Response:** Thanks for this comment by the reviewer. Regarding the English language, we proofread the manuscript for possible vague points and grammatical errors, including those mentioned by the respected reviewer. We finally used Grammarly as an English language application to find possible mistakes. If there are still points for improvement, we would be so happy to have the respected reviewer comment.

Reviewer #6

Abstract

Authors provide urinary concentrations adjusted for creatinine. Authors should specify this so reader understands difference between concentrations in units of µg/L and µg/gcrt
Introduction
Line 94 – "when used in large quantities" – risk from pesticides is related to level of exposure and not quantities used.
Line 97 – different pesticides have different toxic properties. Authors should report toxic properties based on pesticide group (organophosphates, pyrethroids, etc) and not all pesticides together
General – introduction should include more information on status of registration of pesticides in Iran. Is chlorpyrifos approved for use on food crops? Do you have data on amount used? In EU for example this pesticide is no longer approved for plant protection.

**Response:** Thanks for this constructive comment by the reviewer, we revised the introduction section thoroughly and tried to address all the comments.

Sampling
Was study population adults? Children? Although this appears in Table 1 authors should state in text that study population was adults
Were there questions in the questionnaire about diet? Distance of home from agricultural fields? If not this should be mentioned in limitations of study

**Response**: Thank you for your valuable comment. The study population is added to the Material and Method section (adults of age 25 and above).

There were a complete questionnaire about diet (Food Frequency Questionnaire (FFQ)) but no question about distance from agriculture fields, because the study population were from urban areas with significant distance from agriculture fields.

Results
Lines 203-210 – findings of less interest to reader in context of this study

Response: Thank you for this valuable comment. We reduced these parts and referred the exact numbers to the tables for improving readability. Corrected paragraph: “Men were taller and weighed more than women, whereas women had higher BMI and body fat percentages. Detailed statistics are provided in Table 4. Body muscle percentage was higher in men (32.00 ± 4.41 vs. 24.97 ± 2.68). Moreover, the visceral fat percentage was higher in men compared to women (9.17 ± 3.79 vs. 7.38 ± 2.45). Finally, in the comparison between the resting metabolism of men and women, men had a higher resting metabolism (1669.18 ± 210.64 Cal vs. 1395.31 ± 150.22 Cal).”

Line 244 – does this mean people with higher visceral fat had lower levels of TCPy?
**Response: Thank you for this comment. Exactly. As the beta is negative, higher visceral fat is associated with lower TCPy levels.**

Discussion
For readers to understand the findings, it is important to present study results in comparison to current studies in general populations from different countries worldwide. Table 8 includes mostly studies from the US, and many studies in occupational populations. I would encourage authors to look for more recent data emerging from HBM4EU study. Data is available on HBM4EU dashboard. Comparison of results for population in Iran should be compared to current studies (not from 1990s) and not from occupational studies.

**Response:** We want to sincerely thank the reviewer for evaluating our manuscript and providing great comments that improve our manuscript. We provided studies and comparisons with HBM4EU in the discussion section, as the reviewer asked: *“The results of our study are also comparable with the studies from the European Human Biomonitoring Initiative (HBM4EU) which prioritizes pesticides and their exposure (38). These studies should follow specific rules designed by HBM4EU including a minimal number of participants, a specific time period, a pre-designed set of variables, and reporting guidelines. Based on the data provided by this project, Cyprus had a high P50 for TCPy urine levels among children, while this was Israel for adults (39). However, based on estimates, there has been a drastic reduction in exposure to pesticides among European countries from 2016 (39). Regarding the associations between demographic features and pesticide levels, TCPy was shown to be associated with higher education levels and lower BMI levels which is in contrast with our findings (40-42). The difference might be due to a lack of significant difference in terms of diet and Chlorpyrifos exposure among different educational levels in Iran. The country could benefit from a large database such as those in HBM4EU in order to have a better picture of current exposure among different populations and regions.”*

Also, it is worth mentioning results in context of risk assessment. How do results compare to risk based HBM thresholds? See <https://www.sciencedirect.com/science/article/pii/S143846392300010X#bib105>

**Response:** Thanks for this comment. We added a comparison with this study: *“Our findings also showed lower levels of TCPy in the Iranian population, compared to the mean and P95 designed by HBM4EU-aligned studies (43). The country could benefit from a large database such as those in HBM4EU in order to have a better picture of current exposure among different populations and regions.”*

Line 295 – why do you think exposure levels were higher in Kermanshah region? Previous studies have shown that diet is major source of exposure to Chlorpyrifos. Is it possible that there are dietary differences between regions? Or do certain regions have more agriculture and possible drift exposure of the general population?

**Response:** Thanks for this comment. We added some possible explanations for these findings, highlighted in the discussion section: *“The underlying reasons for these differences might be differences in dietary patterns and agricultural methods in different superclusters. The importance of diet in Chlorpyrifos exposure stems from the fact that in previous studies, high dietary fat intake increased the bioavailability of chlorpyrifos (38).”*

In policy discussion – it is worth mentioning that chlorpyrifos has been phased out in many countries, see recent decisions in EU and US.

**Response:** Thanks for this accurate comment. We added that to our discussion: *“Interestingly, chlorpyrifos has been phased out in many countries including some in Europe and America (45). A similar strategy could also be implemented in Iran.”*
