# Supplementary material for: Reference values of urinary metabolites of organophosphate in healthy Iranian adults
Source: PLoS One. 2025 Jun 11;20(6):e0325037. doi: 10.1371/journal.pone.0325037 (PMC12157253; doi:10.1371/journal.pone.0325037)
Supplement: Attachment — Submitted filename: Response to comments- (2) (Autosaved).docx [file pone.0325037.s006.docx]

**Manuscript number** PONE-D-24-11112R2

**Manuscript title:** Reference Values of urinary metabolites of organophosphate in healthy Iranian adults

**Dear Editor-in-chief,**

Our team appreciates the editor and the reviewers for their observations and comments on the manuscript. We have fulfilled their comments and suggestions and wish to submit a revised version of the manuscript for further consideration in the journal. Changes in the revised version of the manuscript are highlighted in green color. Below, we also provide a point-by-point response explaining how we have addressed each of the reviewer and editors’ comments.

Yours sincerely,

**Prof. Alireza Mesdaghinia**

Review Comments to the Author

Please use the space provided to explain your answers to the questions above. You may also include additional comments for the author, including concerns about dual publication, research ethics, or publication ethics. (Please upload your review as an attachment if it exceeds 20,000 characters).

**Reviewer #2:**

1. In the abstract section, the following sentences should be placed after the aim of the article: "We measured metabolites for Chlorpyrifos (TCP: 2-isopropyl-4-methyl-6-hydroxypyrimidine), Diazinon (IMPY: 2-isopropyl-4-methyl-6-hydroxypyrimidine), and Malathion (Malathion dicarboxylic acid) in 490 healthy Iranian adults. Additionally, we recorded age, gender, wealth index, and body composition parameters including body fat, muscle mass, visceral fat, and BMI. Fasting urine sampling, along with body composition and demographic measurements, were conducted. Urine samples were subsequently analyzed."

**Response**: Thank you for your valid comment. This part is removed from the first paragraph and is added after the aim of the study.

2. What do you mean by "Our analysis included 300 ml of morning urine collected from participants on the fourth sampling day"?

**Response**: Thank you for pointing out the unclear sentence. Since this manuscript is part of a larger study on food, water, and air intake relation with pollutants in urine in the Iranian population, the sampling of intakes happened for three days and on the fourth day the urine sample was collected. This information is not relevant to this manuscript though. Therefore, the sentence is revised as “The urine sample of 300 ml fasting urine was collected from participants”

3. Please correct Table 3, as the data presented is unclear. It is essential to apply appropriate statistical tests and analyses for comparing the data effectively.

**Response**: Thank you for this comment, using the family assets questionnaires, a principal component analysis was implemented to create an index showing the wealth status of each family. The created index, then was categorized to 5 different levels from the poorest (Q1) to wealthiest (Q5). Table 3 shows the number of participants in each province and based on each of wealth level (Q1-Q5).

4. In Tables 4, 5, and 6, please utilize a means comparison test to analyze the differences among the various variables, along with the corresponding P-values.

**Response**: Thanks for this constructive comment by the reviewer. Corrected, the necessary explanations have been added to the text and tables.

**Reviewer #6:**

Authors should revise Table 8 to include studies that are more recent. Authors should consider removing occupational studies from this table. Authors mention HBM4EU study but should present data as well (perhaps in Table 8)

**Response**: We want to deeply thank the reviewer for this comment, as the referee rightly asked, we revised the table based on this comment and added the relevant studies to the table.

1. Dalsager L, Fage-Larsen B, Bilenberg N, Jensen TK, Nielsen F, Kyhl HB, Grandjean P, Andersen HR. Maternal urinary concentrations of pyrethroid and chlorpyrifos metabolites and attention deficit hyperactivity disorder (ADHD) symptoms in 2-4-year-old children from the Odense Child Cohort. Environ Res. 2019 Sep;176:108533. doi: 10.1016/j.envres.2019.108533. Epub 2019 Jun 11. PMID: 31229776.
2. Pirard C, Remy S, Giusti A, Champon L, Charlier C. Assessment of children's exposure to currently used pesticides in wallonia, Belgium. Toxicol Lett. 2020 Sep 1;329:1-11. doi: 10.1016/j.toxlet.2020.04.020. Epub 2020 May 1. PMID: 32371136.
3. Yusà V, F Fernández S, Dualde P, López A, Lacomba I, Coscollà C. Exposure to non-persistent pesticides in the Spanish population using biomonitoring: A review. Environ Res. 2022 Apr 1;205:112437. doi: 10.1016/j.envres.2021.112437. Epub 2021 Nov 26. PMID: 34838757.
4. Rodzaj W, Wileńska M, Klimowska A, Dziewirska E, Jurewicz J, Walczak-Jędrzejowska R, Słowikowska-Hilczer J, Hanke W, Wielgomas B. Concentrations of urinary biomarkers and predictors of exposure to pyrethroid insecticides in young, Polish, urban-dwelling men. Sci Total Environ. 2021 Jun 15;773:145666. doi: 10.1016/j.scitotenv.2021.145666. Epub 2021 Feb 6. PMID: 33596511.
5. Faniband MH, Norén E, Littorin M, Lindh CH. Human experimental exposure to glyphosate and biomonitoring of young Swedish adults. Int J Hyg Environ Health. 2021 Jan;231:113657. doi: 10.1016/j.ijheh.2020.113657. Epub 2020 Oct 30. PMID: 33130428.

Line 325 - there have been studies showing toxicity of chlorpyrifos at levels lower than those do that inhibit cholinesterase. Consider citing more recent studies here

**Response**: Thank you for your comment. **Line 324**, updated references are added to the text.

Burke RD, Todd SW, Lumsden E, Mullins RJ, Mamczarz J, Fawcett WP, Gullapalli RP, Randall WR, Pereira EFR, Albuquerque EX. Developmental neurotoxicity of the organophosphorus insecticide chlorpyrifos: from clinical findings to preclinical models and potential mechanisms. J Neurochem. 2017 Aug;142 Suppl 2(Suppl 2):162-177. doi: 10.1111/jnc.14077. PMID: 28791702; PMCID: PMC5673499.

Wołejko E, Łozowicka B, Jabłońska-Trypuć A, Pietruszyńska M, Wydro U. Chlorpyrifos Occurrence and Toxicological Risk Assessment: A Review. Int J Environ Res Public Health. 2022 Sep 26;19(19):12209. doi: 10.3390/ijerph191912209. PMID: 36231509; PMCID: PMC9566616.

Line 327 - "Studies in China and the U.S. reported higher pesticide poisoning rates in women than men" - this does not seem related to low level exposure (which is the topic of the current study) but perhaps suicide attempts? consider taking this statement out

**Response**: Thank you for raising this valid comment in the **line 372**. The statement about higher pesticide poisoning rates in women from studies in China and the U.S. does appear to be about acute pesticide poisoning, is a different issue that often involves intentional exposure or occupational accidents. We omitted the sentence from our text.

*.*
